# Regulation of leptin receptor‐expressing neurons in the brainstem by TRPV1

**DOI:** 10.14814/phy2.12160

**Published:** 2014-09-28

**Authors:** Andrea Zsombok, Yanyan Jiang, Hong Gao, Imran J. Anwar, Kavon Rezai‐Zadeh, Courtney L. Enix, Heike Münzberg, Andrei V. Derbenev

**Affiliations:** 1 Department of Physiology, School of Medicine, Tulane University, New Orleans, Louisiana; 2 Neuroscience Program, School of Science and Engineering, Tulane University, New Orleans, Louisiana; 3 Central Leptin Signaling, Pennington Biomedical Research Center, LSU System, Baton Rouge, Louisiana

**Keywords:** DMV, leptin, patch‐clamp, TRPV1

## Abstract

The central nervous system plays a critical role in the regulation of feeding behavior and whole‐body metabolism via controlling the autonomic output to the visceral organs. Activity of the parasympathetic neurons in the dorsal motor nucleus of the vagus (DMV) determines the vagal tone and thereby modulates the function of the subdiaphragmatic organs. Leptin is highly involved in the regulation of food intake and alters neuronal excitability of brainstem neurons. Transient receptor potential vanilloid type 1 (TRPV1) has also been shown to increase neurotransmission in the brainstem and we tested the hypothesis that TRPV1 regulates presynaptic neurotransmitter release to leptin receptor‐expressing (LepRb^EGFP^) DMV neurons. Whole‐cell patch‐clamp recordings were performed to determine the effect of TRPV1 activation on excitatory and inhibitory postsynaptic currents (EPSC, IPSC) of LepRb^EGFP^ neurons in the DMV. Capsaicin, a TRPV1 agonist increased the frequency of miniature EPSCs in 50% of LepRb^EGFP^ neurons without altering the frequency of miniature IPSCs in the DMV. Stomach‐projecting LepRb^EGFP^ neurons were identified in the DMV using the transsynaptic retrograde viral tracer PRV‐614. Activation of TRPV1 increased the frequency of mEPSC in ~50% of stomach‐related LepRb^EGFP^ DMV neurons. These data demonstrate that TRPV1 increases excitatory neurotransmission to a subpopulation of LepRb^EGFP^ DMV neurons via presynaptic mechanisms and suggest a potential interaction between TRPV1 and leptin signaling in the DMV.

## Abstract (precis)

e12160

Our data demonstrate that TRPV1 is involved in the regulation of a subpopulation of leptin receptor‐expressing neurons in the dorsal motor nucleus of the vagus via presynaptic mechanisms.

## Introduction

Leptin is largely involved in the regulation of food intake and energy expenditure via activating central neural circuits (Elmquist 1998; Frederich et al. 1995; Heymsfield et al. 1999; Leshan et al. 2006; Smedh et al. 1998). Leptin receptors (LepRb) have been identified in many different brain areas including the hypothalamus and the dorsal vagal complex which consists of the area postrema, the nucleus of the solitary tract (NTS) and the dorsal motor nucleus of the vagus (DMV) (Elmquist et al. 1998; Grill et al. 2002; Li et al. 2007; Myers et al. 2009; Patterson et al. 2011; Schwartz and Moran 2002; Shioda et al. 1998; Williams et al. 2009; Williams and Smith 2006). Application of leptin inhibits a subset of DMV neurons, including stomach‐related neurons via ATP‐dependent K^+^ channels (Williams et al. 2007), and excites another subset of DMV neurons (Li et al. 2007). Leptin also decreased glutamatergic neurotransmission in the DMV (Williams et al. 2007) and these mechanisms may underlie the reduced food ingestion response to leptin administration in the dorsal vagal complex (Grill et al. 2002). The dorsal vagal complex largely controls homeostatic functions by integrating information from direct neural projections and from signals relevant to food intake and metabolic status (e.g., leptin) and the parasympathetic motor efferents originating from DMV neurons transfer the signals to subdiaphragmatic organs and thus influence many visceral functions (Babic et al. 2012; Browning and Travagli 2010; Travagli et al. 2006). The activity of DMV neurons is modulated by direct alteration of the cell body (postsynaptic effect) and by synaptic inputs (presynaptic effect), and influenced by metabolic signals (Holmes et al. 2009; Hosoi et al. 2002; Peters et al. 2006a,b; Wan et al. 2007). Therefore identification of receptors, which are able to alter synaptic inputs, is significant due to their ability to modulate the parasympathetic control of homeostatic functions.

Transient receptor potential vanilloid type 1 (TRPV1), the ligand‐gated nonselective cation channel has been shown to modulate synaptic inputs to DMV neurons and thus may influence visceral functions (Derbenev et al. 2006; Zsombok et al. 2011a). Activation of TRPV1 increases neurotransmission to parasympathetic DMV neurons (Derbenev et al. 2006; Zsombok et al. 2011a), and TRPV1 has also been shown to enhance glutamatergic neurotransmission to preautonomic neurons in the hypothalamus and thereby may play a role in controlling autonomic output to various visceral organs (Gao et al. 2012). Since, TRPV1 is an important synaptic regulator of preautonomic neurons both in the hypothalamus and brainstem, leptin plays a significant role in energy homeostasis, and the expression of leptin receptors in the dorsal vagal complex is established we tested the hypothesis that TRPV1 increases neurotransmission to leptin receptor‐expressing (LepRb^EGFP^) neurons in the DMV. Whole‐cell patch‐clamp recordings were conducted from LepRb^EGFP^ DMV neurons and the effect of acute activation of TRPV1 was assessed on synaptic responses in DMV neurons, including subsets of stomach‐related motor neurons.

## Materials and Methods

### Animals

Homozygous breeding pairs of LepRb^Cre^; B6;129‐*Gt(ROSA)26Sor*^*tm2Sho*^/J mice or heterozygous breeding pairs of LepRb^Cre^; B6;129S‐*Gt(ROSA)26Sor*^*tm38(CAG‐GCaMP3)Hze*^/ J mice were used to generate male and female experimental animals with green fluorescent protein expression in LepRb neurons (LepRb^EGFP^ mice) as described elsewhere Leshan et al. (2006); Tian et al. (2009); Zhang et al. (2011). Breeding was performed at Pennington Biomedical Research Center (Baton Rouge, LA), while all experiments were performed at Tulane University (New Orleans, LA). Mice were housed in the vivarium under 12 h light – 12 h dark cycle with food and water available ad libitum. Experiments were performed following the guidelines of the National Institutes of Health Guide for the Care and Use of Laboratory Animals and approved by Pennington Biomedical Research Center's and Tulane University's Institutional Animal Care and Use Committee.

### Injection of PRV‐614

In a set of mice (*n* = 5) retrogradely transported pseudorabies viral vector (PRV‐614, supplied by CNNV Virus Center, Pittsburgh, PA) that expresses red fluorescent protein (RFP) was used to identify stomach‐related neurons as previously described in detail Boychuk et al. (2013); Derbenev et al. (2004); Glatzer et al. (2003). Briefly, under anesthesia, the stomach was exposed and ~2 *μ*L of PRV‐614 was injected into the greater curvature of the stomach fundus using a Hamilton syringe fitted with a 26‐gage needle. The animals were maintained in a biosafety level 2 facility up to 72 h post‐injection.

### Brain slices preparation

Animals were decapitated under anesthesia and acute brainstem slices were prepared and immersed in ice‐cold oxygenated artificial cerebrospinal fluid (aCSF) containing the following (in mmol/L): 124 NaCl, 26 NaHCO_3_, 1.4 NaH_2_PO_4_, 11 glucose, 3 KCl, 1.3 MgCl_2_, 1.5 CaCl_2_, pH 7.3–7.4. Transverse brainstem slices containing the DMV (300 *μ*m) were made using a vibratome. The slices were stored in a holding chamber at 34–36°C, and then transferred to a recording chamber mounted on a fixed stage under an upright microscope (Nikon FN1, Nikon Instruments Inc., Melville, NY).

### Whole‐cell patch‐clamp recordings

Whole‐cell patch‐clamp recordings were performed at 34–36°C on EGFP‐positive LepRb neurons and on stomach‐related (red) LepRb^EGFP^ neurons throughout the DMV identified under 40× water‐immersion objective (N.A = 0.8). Epifluorescence was used to identify EGFP and/or RFP containing neurons and infrared illumination and differential interference contrast optics (IR‐DIC) to target specific cells. For whole‐cell patch‐clamp recordings, electrodes (3–7 MΩ) were filled with a solution containing the following (in mmol/L): 130 K^+^ or Cs^+^ gluconate, 10 HEPES, 5 EGTA, 1 NaCl, 1 MgCl_2_, 1 CaCl_2_, 3 KOH or CsOH, 2–3 Mg‐ATP, 0.2% biocytin, pH 7.3–7.4. Electrophysiological signals were recorded using an Axoclamp 700B amplifier (Molecular Devices, Sunnyvale, CA) and acquired by pClamp (Molecular Devices). Excitatory postsynaptic currents (EPSCs) were recorded at −60 mV, whereas inhibitory postsynaptic currents (IPSCs) were recorded at −10 mV without additional inhibitors. Synaptic currents were analyzed offline using pClamp or MiniAnalysis (Synaptosoft Inc., Decatur, GA). Tetrodotoxin (TTX; 1 *μ*mol/L; Tocris Bioscience, R&D Systems Inc., Minneapolis, MN) in the bath solution was used to block action potentials and monitor miniature IPSCs (mIPSCs) and EPSCs (mEPSCs). Murine leptin (300 nmol/L; PeproTech Inc., Rocky Hill, NJ) or capsaicin (1 *μ*mol/L; R&D Systems) were bath applied and continuous recordings were conducted.

### Statistical analysis

Recordings were analyzed with pClamp or MiniAnalysis. Continuous recordings of EPSCs and IPSCs have been conducted before and after drug application and the data were analyzed in 2 min epochs. The effects of drug on PSC frequency and amplitude were analyzed within individual cells using the Kolmogorov–Smirnov test by comparing 2 min epochs before and after drug application (Boychuk et al. 2013; Gao et al. 2012). The effects of drug across the neuron groups were analyzed using paired *t*‐test. Significance was set *P* < 0.05, numbers were reported as mean ± standard error (SEM).

## Results

### Functional expression of LepRb in the DMV

**Figure 1.**
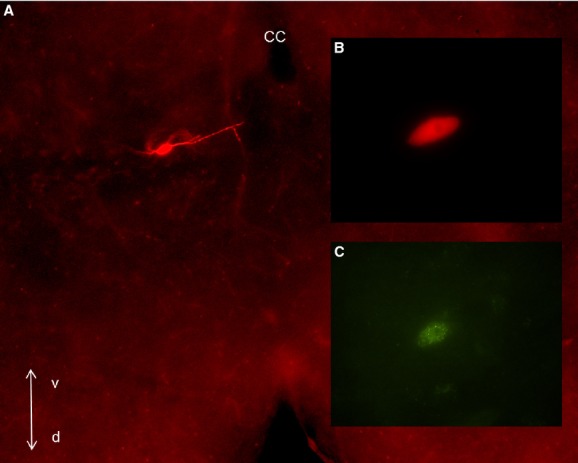
Visualization of a recorded LepRb^EGFP^ neuron in the DMV. (A) Brainstem section (300 *μ*m) following identification of the biocytin labeling with avidin‐Texas Red conjugate (10×). (B) Enlarged image of the soma of the recorded neuron shown in A (100×). (C) Same neuron indicating that the recording was conducted from a LepRb^EGFP^ neuron. cc: central canal, v: ventral; d: dorsal.

**Figure 2.**
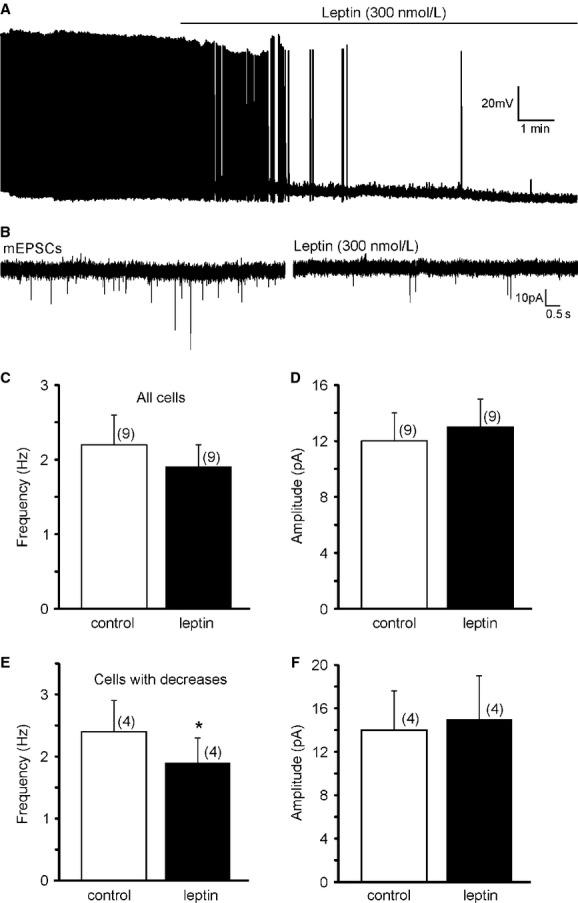
Leptin regulates the resting membrane potential and excitatory neurotransmission in LepRb^EGFP^ neurons of the DMV. (A) Example of leptin caused hyperpolarization and decreased firing rate in a subset of LepRb^EGFP^ neurons. (B) Continuous recordings of mEPSCs demonstrate that leptin (300 nmol/L) decreases the frequency of mEPSCs in a subset of LepRb^EGFP^ neurons in the DMV. (C) Histogram indicates the overall mEPSC frequency of all recorded cells before and after leptin application. (D) Bath administration of leptin did not alter the amplitude of mEPSCs. (E) Leptin decreased the frequency of mEPSCs in a subset of the recorded LepRb^EGFP^ neurons, without altering the amplitude (F).

Previous findings demonstrated that leptin modulates the membrane potential of DMV neurons (Li et al. 2007; Williams et al. 2007), therefore as an initial step we verified the functional expression of leptin receptors in the LepRb^EGFP^ DMV neurons using whole‐cell patch‐clamp recordings in current‐clamp mode. Leptin receptor‐expressing DMV neurons were identified by their green fluorescent labeling (EGFP) (Fig. 1) and recordings were conducted at resting membrane potential (0 pA injected current). The overall resting membrane potential of the recorded LepRb^EGFP^ neurons was −57.1 ± 4.4 mV (range −43.4 to −71.6 mV, *n* = 9). Bath application of leptin (300 nmol/L) caused hyperpolarization in approximately half of LepRb^EGFP^ neurons (−7.2 ± 3 mV, *n* = 5) and decreased the firing of the recorded DMV neurons (2.1 ± 0.9 Hz vs. 0.8 ± 0.8 Hz) (Fig. 2A), whereas the remaining cells depolarized (5.4 ± 2.4 mV, *n* = 4, not shown). These data confirmed functional expression of LepRb in EGFP‐positive DMV neurons and revealed similar response following leptin application than previously reported Li et al. (2007); Williams et al. (2007).

Previous electrophysiological studies from the brainstem also indicated that leptin decreases excitatory neurotransmission in the DVC (Williams and Smith 2006; Williams et al. 2007). Neurons were voltage clamped at −60 mV to reveal the effect of leptin on mEPSC frequency in LepRb^EGFP^ neurons. The average frequency of mEPSCs was 2.2 ± 0.4 Hz before and 1.9 ± 0.3 Hz after application of leptin (*n* = 9) (Fig. 2C). The average amplitude did not change after leptin application (12 ± 2 pA vs. 13 ± 2 pA, *n* = 9) (Fig. 2D). Analysis with Kolmogorov–Smirnov test to determine the response of individual cells to leptin revealed similar findings shown in a previous study by Williams et al. (2007). Leptin decreased the frequency of mEPSCs in a subset (four out of nine) of the recorded LepRb^EGFP^ neurons (Fig. 2B). The frequency of mEPSCs was 2.4 ± 0.5 Hz (range 1.6–3.8 Hz, *n* = 4) before and 1.9 ± 0.4 Hz (range 1.4–3.2 Hz) after application of leptin (*P* < 0.05) (Fig. 2E), but there was no change in the amplitude (14 ± 4 pA vs. 15 ± 4 pA, *n* = 4) (Fig. 2F). The frequency of mEPSCs in the remaining five recorded LepRb^EGFP^ cells was unaltered (2.0 ± 0.6 Hz vs. 1.9 ± 0.6 Hz, *n* = 5). There was no significant change in the amplitude (10.8 ± 1.8 pA vs. 11.0 ± 2.4 pA). These data suggest that LepRb is functional in the EGFP‐labeled neurons and leptin reduces excitatory neurotransmission to a subpopulation of LepRb^EGFP^ neurons in the DMV.

### Capsaicin effect on excitatory neurotransmission to LepRb^EGFP^ neurons

**Figure 3.**
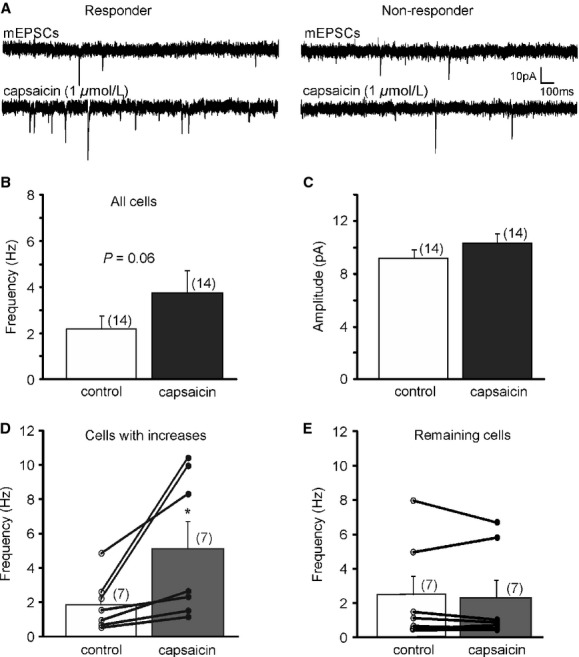
Activation of TRPV1 controls excitatory neurotransmission in a subset of LepRb^EGFP^ neurons in the DMV. (A) Continuous recordings of mEPSCs following capsaicin application revealed two subsets (responder [left] and nonresponder [right]) of LepRb^EGFP^ neurons. (B–C) Capsaicin did not significantly alter the overall average frequency (B) and amplitude (C) of mEPSCs in LepRb^EGFP^ neurons. (D) Capsaicin increased mEPSC frequency in a subset (50%) of LepRb^EGFP^ neurons, whereas there was no change in the remaining cells (Kolmogorov–Smirnov test) (E).

TRPV1 has been shown to increase excitatory and inhibitory neurotransmission in the brainstem including in stomach‐related DMV neurons (Anwar and Derbenev 2013; Derbenev et al. 2006). Leptin regulates feeding behavior and digestion and we tested the hypothesis that TRPV1 increases excitatory neurotransmission in LepRb^EGFP^ DMV neurons. To examine the effect of capsaicin (1 *μ*mol/L), a TRPV1 agonist, on excitatory neurotransmission, LepRb^EGFP^ neurons were voltage clamped at −60 mV and mEPSCs were continuously recorded (Fig. 3A). The average frequency of mEPSCs was 2.2 ± 0.6 Hz (range 0.4–7.9 Hz, n=14) before and 3.7 ± 0.9 (range 0.5–10.3 Hz) after application of capsaicin (*P* = 0.06, *t*‐test paired) (Fig. 3B). There was no change in the average amplitude before and after capsaicin application (9.2 ± 0.6 pA vs. 10.3 ± 0.7 pA; *n* = 14) (*P* > 0.05). Analysis of individual cells with Kolmogorov–Smirnov test revealed two populations of neurons, responders (50%) and nonresponders (50%) (Fig. 3A,D,E). The average mEPSC frequency of the responder group was 1.9 ± 0.6 Hz (range 0.5–4.8 Hz, *n* = 7). Bath application of capsaicin (1 *μ*mol/L) significantly increased mEPSC frequency to 5.1 ± 1.5 Hz (range 1.1–10.4 Hz; 173 ± 43%) (*P* < 0.05). The average amplitude of these neurons were not altered by TRPV1 activation (9.4 ± 1.0 pA vs. 11.9 ± 0.8 pA, *n* = 7) (*P* > 0.05). In contrast to the responder group, capsaicin did not change the frequency of mEPSCs in the remaining 50% of the recorded LepRb^EGFP^ neurons. The average frequency of mEPSCs was 2.4 ± 1.1 Hz (range 0.4–7.9 Hz; *n* = 7) before and 2.3 ± 1.0 Hz (range 0.5–6.7 Hz; −4 ± 10%) following capsaicin application (*P* > 0.05). There was no change in the average of amplitude (8.9 ± 0.8 pA vs. 8.7 ± 0.9 pA).

**Figure 4.**
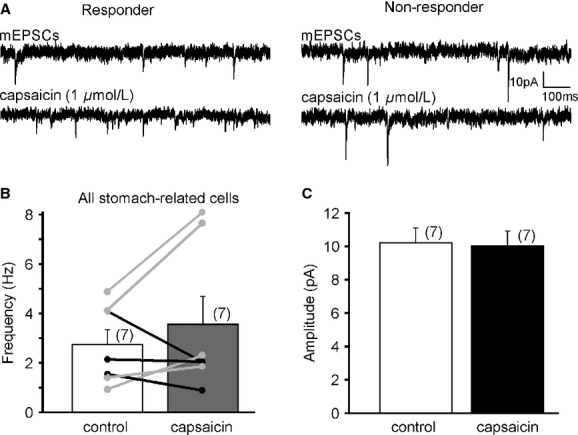
TRPV1 activation increases mEPSC frequency in a subset of stomach‐related LepRb^EGFP^ neurons in the DMV. (A) Continuous recordings of mEPSCs before and after capsaicin application demonstrate responder (traces on the left) and nonresponder (traces on the right) subsets of stomach‐related LepRb^EGFP^ neurons. (B) Graph shows the overall frequencies and the individual responses to TRPV1 activation. Light gray indicates cells which responded with a significant increase to capsaicin application (Kolmogorov–Smirnov test); black indicates cells without significant response. (C) Activation of TRPV1 did not alter the amplitude of mEPSCs in stomach‐related LepRb^EGFP^ neurons.

Because leptin is highly involved in the regulation of feeding we tested whether the neurons responding to capsaicin are gastric related. Stomach‐related DMV neurons were identified with PRV‐614 (expressing RFP) and mEPSCs were recorded from stomach‐related LepRb^EGFP^ neurons in the DMV. The PRV labeling observed at ~72 h postinoculation was consistent with previous publications (Glatzer et al. 2003). The average frequency of mEPSCs was 2.7 ± 0.6 Hz (range 0.9–4.8 Hz, *n* = 7) before and 3.6 ± 1.1 Hz (range 0.9–8.1 Hz) after bath application of capsaicin (1 *μ*mol/L), showing an increasing trend but did not reach significance (Fig. 4). There was no difference in amplitude (10.2 ± 0.9 pA vs. 10.0 ± 0.8 pA, *n* = 7). Analysis of the individual cells with Kolmogorov–Smirnov test also revealed two groups of neurons based on their response to capsaicin (Fig. 4B). Four out of the seven recorded cells responded with an increase to capsaicin (2.8 ± 0.9 Hz vs. 4.9 ± 1.7 Hz). These findings suggest that only a subpopulation of gastric‐projecting neurons respond to capsaicin.

### Capsaicin effect on inhibitory neurotransmission to LepRb^EGFP^ neurons

**Figure 5.**
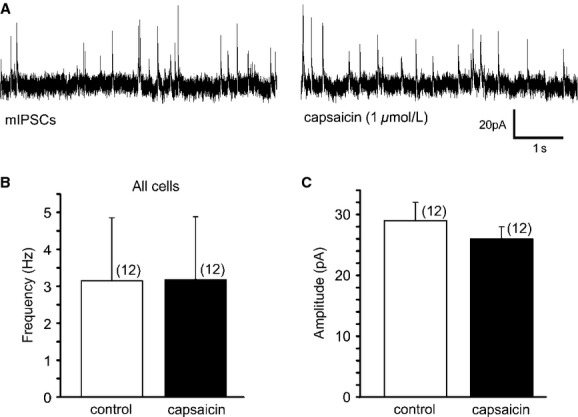
TRPV1 activation did not modulate mIPSCs in LepRb^EGFP^ DMV neurons. (A) Continuous recordings of mIPSCs before and after capsaicin application. (B–C) Application of capsaicin did not alter the overall average frequency (B) and amplitude (C) of mIPSCs.

To determine the effect of capsaicin on inhibitory synaptic activity, neurons were voltage clamped at −10 mV and miniature inhibitory postsynaptic currents (mIPSCs) were examined. The average mIPSC frequency of LepRb^EGFP^ neurons was 3.15 ± 1.7 Hz (range 0.3–21.4 Hz, *n* = 12). Bath application of capsaicin (1 *μ*mol/L) did not change the overall frequency of mIPSCs (3.18 ± 1.7 Hz, range 0.2–21.7 Hz, *n* = 12) (*P* > 0.05); however, three out of the recorded 12 cells showed significant increase with Kolmogorov–Smirnov test. The average amplitude was also unaltered after capsaicin application (29.0 ± 3.0 pA vs. 25.9 ± 2.4 pA, *n* = 12) (*P* > 0.05). These data suggest that TRPV1 activation does not alter the overall inhibitory neurotransmission to LepRb^EGFP^ neurons in the DMV (Fig. 5).

## Discussion

In this study we present novel information about synaptic regulation of leptin receptor‐expressing (LepRb^EGFP^) DMV neurons by TRPV1. Our data demonstrate that activation of TRPV1 increased excitatory neurotransmission in a subset of leptin receptor‐expressing DMV neurons, including stomach‐related LepRb^EGFP^ neurons. On the other hand, TRPV1 activation did not alter the overall inhibitory neurotransmission. Our findings also demonstrated that leptin receptor‐expressing DMV neurons receive synaptic inputs modulated by leptin.

It is important to understand the central mechanisms that control gastrointestinal functions, as a potential target to treat the growing incidence of obesity and eating disorders (Schwartz and Moran 2002; Holmes et al. 2009; Zsombok and Smith 2009). Leptin regulates energy homeostasis through modulation of the autonomic nervous system (Myers et al. 2008; Gautron and Elmquist 2011); however, the synaptic regulation of neurons‐expressing leptin receptors is largely unknown. In general, excitatory and inhibitory synaptic inputs largely control the excitability of neurons. Excitatory inputs arriving to the DMV from a variety of brain areas including the hypothalamus and brainstem, transfer information to the DMV and thus ultimately alter the function of visceral organs (Travagli et al. 2006; Geerling et al. 2010; Babic et al. 2012). Our study revealed the novel information that a subset (approximately half) of leptin receptor‐expressing DMV neurons identified by their EGFP labeling is controlled by TRPV1‐dependent excitatory neurotransmitter release. We can speculate that activation of TRPV1 may modulate the effect of leptin on food intake; however, to prove this scenario requires detailed in vivo experiments, which could be subjects of future studies. Also, the phenotype of the capsaicin‐responsive LepRb^EGFP^ neurons remains an intriguing question. Most of the DMV neurons projecting to the stomach are cholinergic; however, DMV neurons could be divided into subpopulations based on their response to neurotransmitters, hormones, or metabolic signals including insulin, CCK, serotonin, NPY, and others (Browning and Travagli 2009; Mussa et al. 2010; Blake and Smith 2012). Therefore, further detailed investigations would be necessary to determine characteristics of capsaicin‐responsive LepRb DMV neurons.

As an initial step we confirmed that LepRb^EGFP^ neurons in the DMV respond to leptin. Application of leptin caused both hyperpolarization and depolarization in the LepRb^EGFP^ DMV neurons. This is consistent with previous electrophysiological observations from DMV neurons describing both hyperpolarization and depolarization following leptin administration (Li et al. 2007; Williams et al. 2007). The hyperpolarization was determined as activation of membrane conductance, while the depolarization may be due to inhibition of a tonically activated potassium conductance as explained earlier (Li et al. 2007; Williams et al. 2007). The study by Li and his coworkers (Li et al. 2007) showed no significant difference in the passive membrane properties of the DMV neurons responding with hyperpolarization or depolarization to leptin application. Moreover, the reversal potentials of leptin‐induced currents were not significantly different between the groups (Li et al. 2007). In our case, we did not observe significant difference in the resting membrane potentials between neurons responding either with hyperpolarization or depolarization to leptin application.

Previous findings also described decreased spontaneous and miniature EPSC frequencies in approximately half of the DMV neurons following leptin application (Williams et al. 2007). Our data extended these findings and showed that LepRb^EGFP^ neurons receive excitatory signals which are reduced by leptin. Taken together our data confirmed functional expression of leptin receptors in EGFP neurons and also suggest that leptin is able to modulate the excitability of LepRb^EGFP^ DMV neurons through pre‐ and postsynaptic mechanisms.

Our findings revealed that TRPV1 plays a role in controlling the excitability in a subset of LepRb^EGFP^ neurons in the DMV. TRPV1 expression was described in a variety of brain areas including the dorsal vagal complex (Mezey et al. 2000; Cavanaugh et al. 2011), and immunostaining studies from our laboratory were consistent with these findings (Zsombok et al. 2011b). Furthermore, electrophysiological investigations determined presynaptic location of TRPV1 receptors (Derbenev et al. 2006; Zsombok et al. 2011a), and showed that activation of TRPV1 increased both excitatory and inhibitory neurotransmission in DMV neurons of rats (Derbenev et al. 2006). Furthermore, it also has been demonstrated that TRPV1 contributes to asynchronous neurotransmitter release (Peters et al. 2010; Shoudai et al. 2010). In our study we observed increased frequency of mEPSCs, which is consistent with the previous publications. On the other hand, we did not find changes in the overall inhibitory neurotransmission following capsaicin application. This difference can originate from a specific population of the recorded DMV neurons (LepRb‐expressing neurons vs. random DMV neurons), or there is also the possibility that TRPV1‐dependent inhibitory neurotransmission plays less important role in the regulation of LepRb^EGFP^ neurons compared with excitatory neurotransmission. However, to prove this scenario requires further investigations.

Our data also indicate that only a subset (approximately half) of the LepRb‐expressing DMV neurons are controlled by TRPV1. This may suggest that TRPV1 regulates a specific population of LepRb^EGFP^ DMV neurons and we tested whether the responsive neurons are gastric related. Our data revealed that a similar portion of stomach‐related LepRb‐expressing DMV neurons responded to TRPV1 activation. It is possible that LepRb^EGFP^ DMV neurons projecting to another organs (e.g., liver) are also regulated by TRPV1; but further studies are required to determine this scenario. Moreover, future experiments including in vivo activation of TRPV1 in the brainstem could be used to determine the effect of capsaicin on leptin controlled food intake and gain more information about the possible interaction of TRPV1 and leptin.

In summary, our data revealed that a subset of leptin receptor‐expressing DMV neurons, including stomach‐related LepRb^EGFP^ DMV neurons, is controlled by TRPV1. Therefore, it is possible that the TRPV1‐responsive LepRb^EGFP^ neurons may participate in the regulation of more than one particular organ as indicated by the effect of leptin on feeding behavior and glucose homeostasis (Gautron and Elmquist 2011). However, further investigations are required to determine the phenotype and projections of TRPV1 controlled LepRb‐expressing neurons in the DMV.

## Conflict of Interest

The authors declare no conflict of interest.
